# Establishment and functional characterization of an *EGFR*-amplified tumoroid from squamous cell carcinoma of unknown primary

**DOI:** 10.1007/s13577-026-01440-x

**Published:** 2026-08-19

**Authors:** Miki Iwai, Etsuko Yokota, Takuro Yukawa, Kizuki Matsumoto, Takashi Urano, Kazutaka Nakashima, Hiroyoshi Doihara, Nagio Takigawa, Hideyo Fujiwara, Takashi Akiyama, Minoru Haisa, Yoshio Naomoto, Takuya Fukazawa, Tomoki Yamatsuji

**Affiliations:** 1 Kawasaki Medical School, Central Research Institute, Okayama, Japan; 2 Department of General Surgery, Kawasaki Medical School, Okayama, 700-8505 Japan; 3 Department of General Internal Medicine 4, Kawasaki Medical School, Okayama, Japan; 4 Department of Pathology, Kawasaki Medical School, Okayama, Japan; 5 Kawasaki Medical School General Medical Center, Okayama, Japan

**Keywords:** Carcinoma of unknown primary, Tumoroid, Sensitivity test, *EGFR* amplification, Functional precision oncology

## Abstract

Carcinoma of unknown primary (CUP) remains a clinically challenging malignancy with limited treatment options and poor prognosis. Although genomic profiling has identified potentially actionable alterations, optimal therapeutic strategies are often unclear. Here, we report the establishment and characterization of a patient-derived tumoroid from an *EGFR*-amplified CUP and evaluate its utility as a functional model for therapeutic assessment. The patient-derived tumoroid (PDT-CUP#1) was successfully established from resected tumor tissue and maintained in long-term culture. Whole-exome sequencing demonstrated genomic concordance between the PDT-CUP#1 and the original tumor. Quantitative PCR and fluorescence in situ hybridization confirmed *EGFR* amplification. Xenograft tumors derived from PDT-CUP#1 recapitulated the histopathological features of the original lesion. Functional drug testing demonstrated differential sensitivity among EGFR-targeted agents. Whereas EGFR tyrosine kinase inhibition resulted in modest growth suppression, anti-EGFR monoclonal antibodies exhibited moderate antitumor effects, and an EGFR-targeted antibody–drug conjugate showed the most pronounced growth inhibition. Notably, the therapeutic sensitivity observed in the tumoroids was generally consistent with the patient’s clinical response to platinum-based chemotherapy combined with necitumumab. These findings demonstrate that a CUP-derived tumoroid can be successfully established while preserving the key molecular and pathological features of the original tumor. This model provides a platform for functional evaluation of therapeutic vulnerabilities and offers proof-of-concept for integrating patient-derived tumoroids into therapeutic decision-making in CUP.

**Supplementary Information:**

The online version contains supplementary material available at 10.1007/s13577-026-01440-x.

## Introduction

Precision oncology has substantially advanced cancer treatment by enabling therapeutic stratification based on genomic alterations [1]. However, accumulating evidence suggests that genomic profiling alone may not always be sufficient to predict optimal therapeutic responses, particularly when identical genomic alterations are associated with variable sensitivity to distinct drug classes [2–4]. These observations underscore the potential value of complementary approaches that incorporate functional assessment of tumor-specific drug responses [5]. Functional precision oncology, which integrates patient-derived cancer models with systematic drug testing, has emerged as a promising strategy to complement genomic profiling [6].

Patient-derived tumoroids have emerged as valuable tools for precision oncology. In this study, the term tumoroid refers to a long-term expandable three-dimensional tumor model established from patient-derived tumor tissue, consistent with recent literature [7–9]. Their long-term expandability enables the establishment of stable clonal populations and provides reproducible, shareable experimental resources for functional analyses and drug screening.

In our previous studies, we established patient-derived tumoroids from multiple cancer types, including lung and esophageal cancers [9–13], and demonstrated their utility as preclinical models for functional evaluation of responses to molecularly targeted agents. Building upon these findings, we surmised that a similar functional approach might be applicable to carcinoma of unknown primary (CUP), a clinically challenging disease with limited therapeutic guidance.

CUP, a metastatic disease for which the primary site cannot be identified despite comprehensive diagnostic evaluation, accounts for approximately 2–4% of all cancers and is associated with high mortality [14, 15]. Most patients fall into the poor-risk category, characterized by aggressive disease biology, limited response to empiric chemotherapy, and a one-year survival rate of approximately 20% [16, 17]. Although advances in molecular profiling have improved diagnostic classification and helped identify potentially actionable alterations, molecularly guided treatment strategies have not consistently improved clinical outcomes in CUP [18, 19]. Therefore, approaches that allow direct evaluation of tumor-specific drug sensitivity may provide additional clinical insight.

In the present study, we established and characterized patient-derived tumoroids from a CUP harboring *EGFR* amplification. We further performed functional drug testing to evaluate differential sensitivity across distinct classes of EGFR-targeted agents and explored their potential utility for therapeutic prioritization in this clinically challenging setting.

## Materials and methods

### Cell lines and culture conditions

Human pulmonary adenocarcinoma cell lines carrying distinct oncogenic alterations were used in this study. *KRAS*-mutant cell lines A549 (CCL-185^™^), H358 (CRL-5807^™^), and H460 (HTB-177^™^); *EGFR*-mutant cell lines H1975 (CRL-5908^™^) and NCI-H3255 (CRL-2882^™^); and the *EML4–ALK* fusion-positive cell line NCI-H2228 (CRL-5935^™^) were obtained from the American Type Culture Collection (ATCC, Manassas, VA, USA). *EGFR*-mutant PC-9 cells (RCB4455) were obtained from the RIKEN BioResource Research Center (RIKEN BRC, Tsukuba, Japan). The *EML4–ALK* fusion-positive cell line H3122 was obtained from the National Institutes of Health (NCI, Bethesda, MD, USA). The human lung squamous cell carcinoma cell lines used in this study were obtained as follows: NCI-H2170 (CRL-5928™) from ATCC; EBC-1 (JCRB0820) and LK-2 (JCRB0829) from the Japanese Collection of Research Bioresources (JCRB) Cell Bank (Osaka, Japan); and EBC-2 and SQ-5 as kind gifts from Dr. Katsuyuki Kiura (Department of Respiratory Medicine, Okayama University Graduate School of Medicine and Dentistry, Okayama, Japan) [20]. A549 cells were maintained in high-glucose Dulbecco’s Modified Eagle Medium supplemented with 10 mM HEPES, 10% heat-inactivated fetal bovine serum, 100 U/mL penicillin, and 100 μg/mL streptomycin. All other cell lines were cultured in RPMI-1640 medium containing the same supplements. Cells were incubated at 37 °C under humidified conditions with 5% CO_₂_. Cell line authentication was performed by short tandem repeat (STR) profiling using the GenePrint^®^ 10 System (Promega, Madison, WI, USA). Routine screening for mycoplasma contamination was performed using a PCR-based detection kit (Takara Bio, Otsu, Japan).

### Patient information, tumor sampling, and tumoroid establishment

A 69 year-old man presented with a palpable mass in the left axilla. Despite a course of antibiotic treatment after initial visit to a local clinic, the mass did not improve, and he was referred to our hospital. He underwent excision of the left axillary mass, which was later confirmed to represent a metastatic lymph node lesion, together with axillary lymph node dissection. The left axillary mass represented a metastatic lymph node lesion of CUP. Tumor tissue obtained from this lesion was used for tumoroid establishment. Patient-derived tumoroids (PDT-CUP#1) were established using a previously reported tumoroid culture system [21]. In addition to the medium optimized for lung cancer [21–23], culture conditions developed for breast and esophageal cancers were also applied [24–26]. Ethical approval for this study was obtained from the Ethics Committee of Kawasaki Medical School (approval no. 3171-8). STR profiling of PDT-CUP#1 tumoroids was performed using the same method as that used for cell line authentication. The patient signed an informed consent form approved by the responsible authority and provided written informed consent in accordance with protocols approved by the Institutional Review Board. The patient provided written informed consent in accordance with protocols approved by the Institutional Review Board.

### Whole-exome sequencing (WES), Sanger sequencing, and fluorescence in situ hybridization (FISH)

WES was performed by Novogene Co., Ltd. (Beijing, China). Genomic DNA libraries were prepared using the Agilent SureSelect Human All Exon V6 kit (Agilent Technologies, Santa Clara, CA, USA) with a hybrid-selection protocol and sequenced on the Illumina NovaSeq X Plus platform using paired-end 2 × 150 bp reads (average insert size, 180–280 bp). Sequence reads were aligned to the human reference genome (GRCh38; GCA_000001405.15) using BWA (v0.7.17). Single-nucleotide variants (SNVs) and short insertions/deletions (Indels) were identified using GATK (v4.3.0), and copy number alterations (CNAs) were analyzed using CNVkit (v0.9.10). Because matched normal DNA was unavailable, variant-calling was performed using the public GATK Panel of Normal s (PON) to reduce germline variants and technical artifacts. Accordingly, germline–somatic discrimination relied on PON-based filtering, and comparative genomic analysis between the parental tumor and the corresponding tumoroid focused primarily on shared sequence variants and shared copy number alterations. Gene-level copy number annotations were generated from the identified CNA segments.

Sanger sequencing and karyotypic evaluation were performed using FISH with an alpha-satellite probe (orange) [9]. The FISH probes for *EGFR* and chromosome 7 centromere (Cen7) (Vysis LSI EGFR SpectrumOrange/CEP 7 SpectrumGreen Probe Set; catalog no. 5J4801_30-191053) were obtained from Abbott Molecular, Inc. (Des Plaines, IL, USA). Table S1 lists the primers used for PCR and Sanger sequencing in this study.

Genomic DNA extracted from PDT-CUP#1 was used for Sanger sequencing at passage nine, WES at passage 10, and FISH analysis at passage 15.

### Copy number assay using quantitative real-time polymerase chain reaction (qPCR)

Genomic DNA extracted from PDT-CUP#1 at passage nine was performed using the QIAamp DNA Mini Kit (QIAGEN). *EGFR* copy number was quantified via qPCR with an EGFR-specific probe (Hs07526740_cn) on a QuantStudio 3 (Thermo Fisher Scientific, Waltham, MA, USA). RNase P was used for normalization, and copy numbers were derived from triplicate reactions using ABI Copy Caller.

### Cell viability assay

Tumoroids at passage nine or earlier were used for all drug screening experiments. PDT-CUP#1, PDT-LUAD#5, and PDT-LUAD#43 tumoroids were seeded into 96-well cell culture plates (IsoPlate^™^−96 TC, 6005070; Revvity, MA, USA) at 3.0 × 10^3^cells per well (n = 6 wells per condition) [9]. H3255 pulmonary adenocarcinoma cells and H2170 lung squamous cell carcinoma cells were seeded at densities of 1.5 × 10^3^ and 2.0 × 10^3^ cells per well, respectively (n = 6 wells per condition). Viability measurements were obtained at 96 h post-treatment using the CellTiter-Glo^®^ 2.0 system (Promega, Madison, WI, USA), and luminescence was measured with a Varioskan LUX multimode reader (Thermo Fisher Scientific). Erlotinib, cetuximab, necitumumab, depatuxizumab mafodotin, and human IgG1 isotype controls were purchased from Selleck Biotechnology (Yokohama, Japan).

### Xenografts inoculated from PDT-CUP#1 tumoroids

PDT-CUP#1 tumoroids at passage 14 were dissociated using TrypLE^™^ Express Enzyme (Thermo Fisher Scientific), and 4.0 × 10^6^ cells were mixed with 50 µL of basement membrane extract type 2 prior to subcutaneous implantation into five-week-old NSG mice. Mice were sacrificed 36 d post-inoculation before tumors reached 2 cm in diameter. All animal studies were approved by the Animal Research Committee of Kawasaki Medical School (approval no. 25–029).

### Immunoblot analysis and immunohistochemistry (IHC)

Tumoroids at passage 11 or earlier were used for immunoblotting. Immunoblotting and IHC were performed as described previously [20]. Table S2 lists the antibodies used in this study.

### Statistical analysis

Group differences were analyzed using an unpaired, two-sided Student’s *t*-test. A *p*-value <0.01 was considered statistically significant. Results are presented as mean ± SD. Cell viability and IC_50_ values were calculated using GraphPad Prism (version 10.0; GraphPad Software, San Diego, CA, USA).

## Results

### Clinicopathological and genomic characterization of the CUP case

**Fig. 1:**
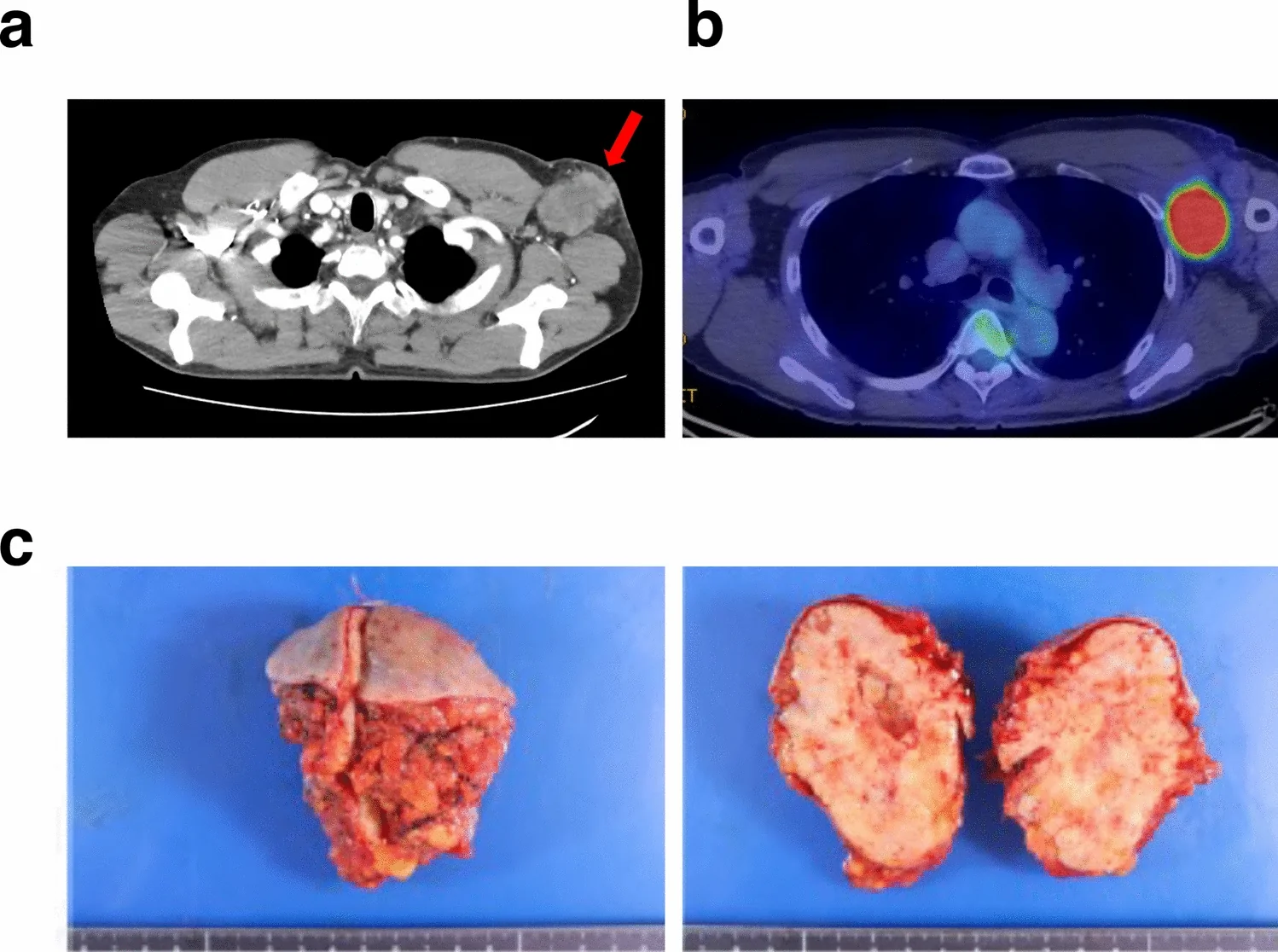
Clinical presentation of the patient with PDT-CUP#1. **a** CT revealed a mass with a maximum diameter of 4.5 cm in the left axillary region (red arrow). **b** PET-CT revealed intense FDG uptake within the mass. **c** Gross appearance of the resected tumor (left panel) and its cut surface (right panel)

Clinical course and sample collection procedure are summarized in the Materials and Methods section. Briefly, a 69 year-old man presented with a left axillary mass that was subsequently diagnosed as CUP. Computed tomography (CT) revealed a 4.5 cm mass in the left axillary region (Fig. 1a), and positron emission tomography-CT (PET-CT) demonstrated intense fluorodeoxyglucose (FDG) uptake within the mass (Fig. 1b). Fine-needle aspiration cytology of the axillary mass led to a diagnosis of squamous cell carcinoma metastasis to the lymph node. Subsequently, for diagnostic and therapeutic purposes, excision of the left axillary mass (Fig. 1c) and axillary lymph node dissection were performed. Postoperative pathology confirmed axillary lymph node metastasis of squamous cell carcinoma; however, the primary site remained unidentified. Contrast-enhanced chest CT revealed no suspicious pulmonary lesions or mediastinal/hilar lymphadenopathy, and PET-CT demonstrated no abnormal FDG uptake other than in the axillary lesion (Fig. S1). Clinical examination revealed no visible skin lesions suggestive of a primary cutaneous malignancy. Histopathological examination of the resected specimen demonstrated neither epidermal dysplasia nor carcinoma in situ within the overlying epidermis, and no histological continuity between the epidermis and the tumor was observed (Fig. S2).

For precision oncology, DNA was extracted from the resected tumor specimen and analyzed using FoundationOne^®^ CDx (Foundation Medicine, Inc.), a clinically validated next-generation sequencing-based comprehensive genomic profiling assay. This test evaluates genomic alterations in 324 cancer-related genes and provides information on clinically relevant biomarkers including microsatellite instability and tumor mutational burden, enabling the identification of potential targeted therapies. Tumor profiling identified five pathogenic mutations and *EGFR* amplification (Table 1).

**Table 1:** Biomarkers and genomic markers findings detected using FoundationOne^®^ CDx

Biomarker findings
HRD (homologous recombination deficiency) signature—negative
Microsatellite status—MS stable
Tumor mutation burden—6 Mut/Mb

Genomic findings
* EGFR* amplification
* STK11* D194N
* TP53* R213Q, R175H
* MAP3K1* S941*
* TERT* promoter -124C>T

### Establishment of tumoroids from CUP

**Fig. 2:**
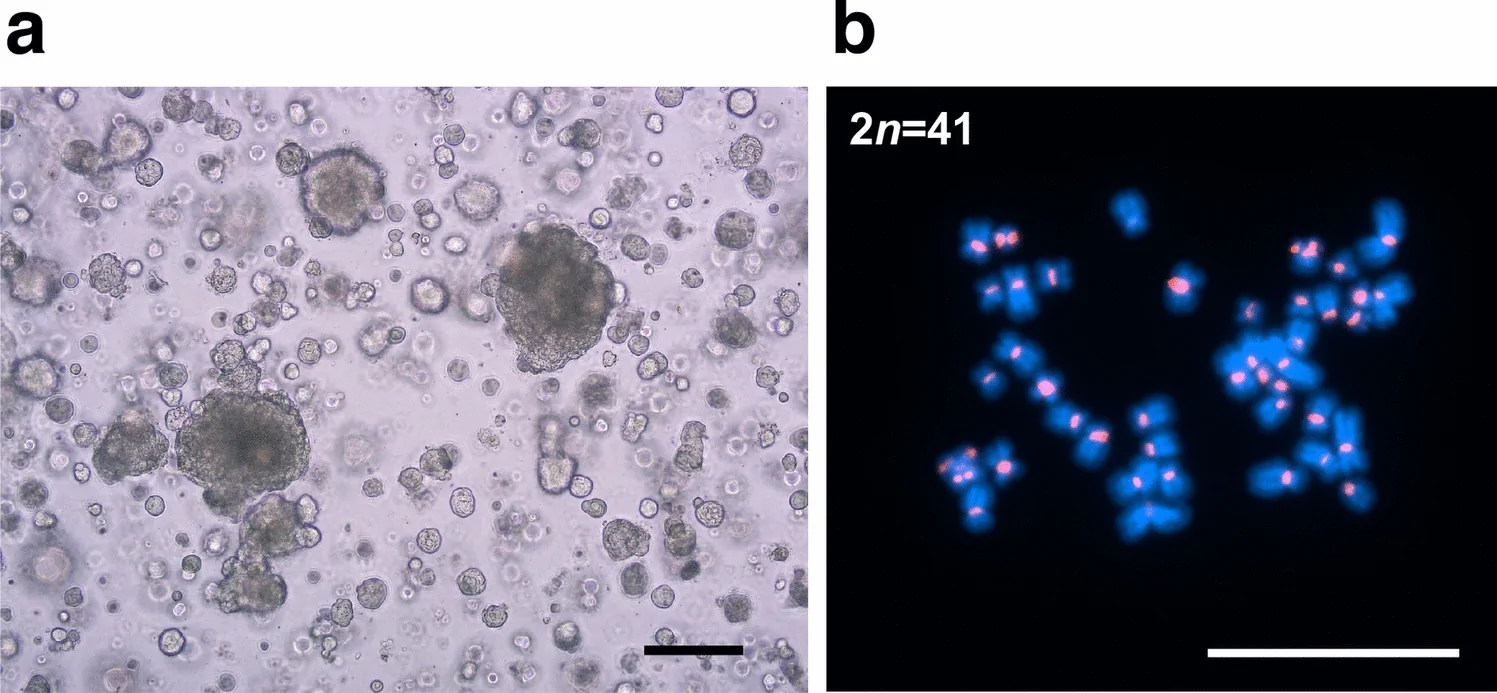
Morphology and karyotyping of PDT-CUP#1 tumoroids. **a** PDT-CUP#1 tumoroids exhibited dense and irregular morphology. Scale bar: 200 μm. **b** FISH analysis of centromeres revealed numerical chromosomal aberrations (2*n*=41) in the tumoroids. Scale bar: 20 μm

We generated a PDT-CUP#1 tumoroid from the resected tumor. Among the multiple culture media tested [21–26], successful establishment was achieved only using the airway organoids (AO) medium or the AO medium supplemented with nutlin-3a [21]. All experiments described in this study were performed using the tumoroids established and maintained under nutlin-3a-free conditions. The tumoroids exhibited irregular and densely packed structures (Fig. 2a). Karyotyping via FISH using an alpha-satellite probe, which specifically recognizes centro meric repetitive DNA sequences, demonstrated that PDT-CUP#1 harbored an aneuploid karyotype (2*n* = 41), confirming its successful establishment from cancer (Fig. 2b). The tumoroid was cultured continuously for at least four mo, corresponding to 18 passages.

### Detection of genetic alterations identified using genome panel testing in genomic DNA from PDT-CUP#1 tumoroids

**Fig. 3:**
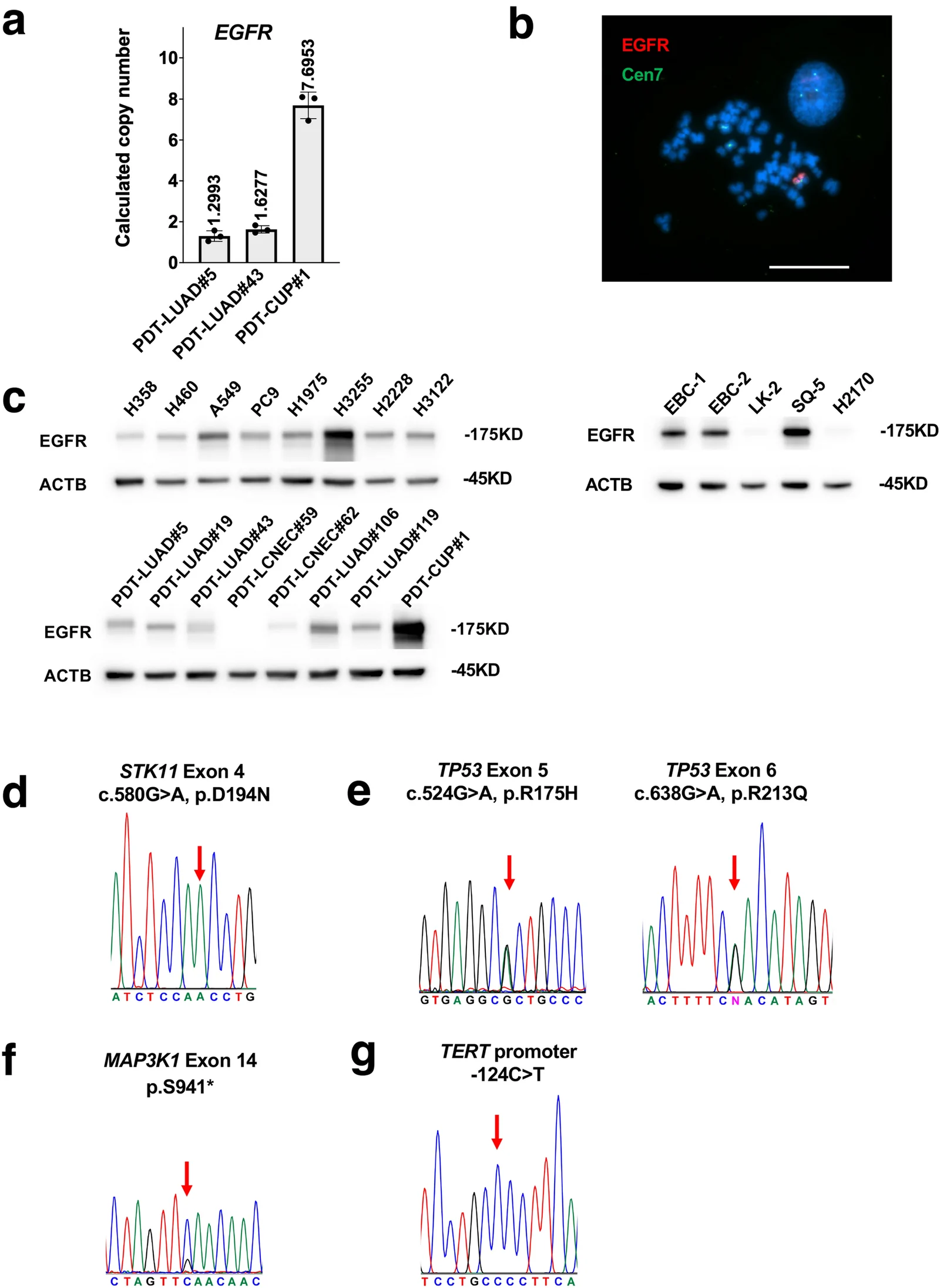
Validation of genetic alterations identified via genome panel testing in PDT-CUP#1 tumoroids. **a** qPCR analysis revealed *EGFR* amplification in PDT-CUP#1 tumoroids. **b** Metaphase FISH revealed *EGFR* amplification on one of the three copies of chromosome 7 in PDT-CUP#1. Unlike in H3255 cells, where the *EGFR* probe detected both amplified and non-amplified *EGFR* loci (Fig. S4b), no *EGFR* signals were detected for the remaining two chromosome 7 copies in PDT-CUP#1, indicating deletion of the *EGFR* locus on these chromosomes. Scale bar: 20 μm. **c** Immunoblot analysis revealed that PDT-CUP#1 tumoroids expressed the highest levels of EGFR among all lung cancer cell lines and patient-derived tumoroids analyzed. H358, H460, and A549: pulmonary adenocarcinoma cell lines harboring *KRAS* mutations; PC9, H1975, and H3255: pulmonary adenocarcinoma cell lines harboring *EGFR* mutations; H2228 and H3122: pulmonary adenocarcinoma cell lines harboring *EML4–ALK* fusion genes; EBC-1, EBC-2, LK-2, SQ-5, and H2170: human lung squamous cell carcinoma cell lines; PDT-LUAD#5: patient-derived pulmonary adenocarcinoma tumoroids harboring a *BRAF* mutation [9]; PDT-LUAD#19: patient-derived pulmonary adenocarcinoma tumoroids harboring a *TPM3–ROS1* fusion gene [9]; PDT-LUAD#43: patient-derived pulmonary adenocarcinoma tumoroids harboring an *EGFR* mutation that subsequently transformed into small cell lung cancer [9]; PDT-LCNEC#59 and PDT-LCNEC#62: patient-derived pulmonary large-cell neuroendocrine carcinoma tumoroids [11]; PDT-LUAD#106: patient-derived pulmonary adenocarcinoma tumoroids established from malignant pleural effusion; and PDT-LUAD#119: patient-derived pulmonary adenocarcinoma tumoroids harboring an *EML4–ALK* fusion gene [12]. **d–g** Sanger sequencing confirmed *STK11*, *TP53*, *MAP3K1*, and *TERT* promoter mutations in PDT-CUP#1 tumoroids, which were identified using FoundationOne^®^ CDx analysis of DNA extracted from paraffin-embedded parental tumor tissue

To confirm *EGFR* amplification detected using FoundationOne^®^ CDx in PDT-CUP#1 tumoroids, copy number analysis was performed using qPCR. *EGFR* was markedly amplified in genomic DNA extracted from PDT-CUP#1 tumoroids, whereas no significant amplification was observed from PDT-LUAD#5 and PDT-LUAD#43 tumoroids derived from pulmonary adenocarcinoma (Table 1 and Fig. 3a). Amplification was further validated via FISH using an *EGFR*-specific probe. Two of the three copies of chromosome 7 did not give *EGFR* signals, whereas the remaining copy showed marked *EGFR* amplification (Fig. 3b).

WES-based copy number analysis further confirmed high-level *EGFR* amplification in both the original parental tumor and PDT-CUP#1 tumoroids (Fig. S3). These findings are consistent with the FoundationOne^®^ CDx results obtained from the parental tumor and demonstrate that this clinically relevant *EGFR* amplification was faithfully preserved in the tumoroids.

Immunoblotting demonstrated that PDT-CUP#1 tumoroids expressed the highest levels of EGFR protein among the eight types of lung tumoroids and 13 lung cancer cell lines, including H3255 pulmonary adenocarcinoma cells harboring an EGFR exon 21 L858R mutation with *EGFR* amplification (Figs. 3c, S4).

To further evaluate the genomic fidelity of PDT-CUP#1 tumoroids, WES was performed on paired parental tumor tissue and PDT-CUP#1 tumoroids. WES confirmed that the major pathogenic sequence variants identified using FoundationOne^®^ CDx, including *TP53* mutations (p.R175H and p.R213Q), an *STK11* mutation (p.D194N), and a *MAP3K1* mutation (p.S941*), were retained in PDT-CUP#1 tumoroids. In addition, among genes included in the FoundationOne^®^ CDx panel, WES identified additional shared protein-altering variants in *CARD11*, *CD22*, *CUL4A*, *ERG*, *GNAS*, *LTK*, *NF1*, *PRKN*, and *SGK1* (Table 2).

**Table 2:** Protein-altering or splice-site variants in FoundationOne^®^ CDx panel genes shared between the parental tumor and the PDT-CUP#1 tumoroids

Gene	Alteration	Type	Genomic position	Patient WES	PDT-CUP#1 WES	FoundationOne® CDx
*TP53*	p.R213Q	missense	chr17:7,674,893 C>T	Somatic	Shared	Reported
*TP53*	p.R175H	missense	chr17:7,675,088 C>T	Somatic	Shared	Reported
*STK11*	p.D194N	missense	chr19:7,674,893 C>T	Somatic	Detected (auto-called as germline)*	Reported
*MAP3K1*	p.S941*	stopgain	chr5:56,882,022 C>G	Manual review**	Detected (auto-called as germline)*	Reported
*CARD11*	p.A581T	missense	chr7:2,928,611 C>T	Somatic	Shared	Not reported
*CD22*	p.S487G	missense	chr19:35,341,920 A>G	Somatic	Shared	Not reported
*CUL4A*	p.R71Q	missense	chr13:113,210,036 G>A	Somatic	Shared	Not reported
*ERG*	p.P312L	missense	chr21:38,383,632 G>A	Somatic	Shared	Not reported
*GNAS*	p.E57*	stopgain	chr20:58,853,434 G>T	Somatic	Shared	Not reported
*LTK*	p.E633K	missense	chr15:41,504,401 C>T	Somatic	Shared	Not reported
*NF1*	p.C1857W	missense	chr17:31,330,320 T>G	Somatic	Shared	Not reported
*PRKN*	splice-site	splicing	chr6:162,054,177 T>C	Somatic	Shared	Not reported
*SGK1*	p.T383M	missense	chr6:134,170,284 G>A	Somatic	Shared	Not reported

*Automatically classified as germline by the WES variant-calling pipeline because all sequencing reads supported the variant allele

**Not called by the WES variant-calling pipeline but confirmed by manual inspection of BAM files (13 variant-supporting reads among 131 total reads)

“Reported” indicates alterations listed in the clinical FoundationOne^®^ CDx report as pathogenic. “Not reported” indicates that the gene is included in the FoundationOne^®^ CDx panel, but the corresponding variant was not listed in the clinical report

Comprehensive analysis of the WES data further identified 199 shared SNVs/indels across 97 genes (Table S3), indicating that PDT-CUP#1 retained a broad spectrum of somatic alterations present in the parental tumor. Because no matched normal DNA sample was available, the *STK11* and *MAP3K1* variants were initially classified as germline by the automated WES pipeline owing to their high variant allele frequencies. However, manual inspection of the BAM files together with comparison to the FoundationOne® CDx results supported the interpretation of these variants as shared somatic alterations present in both the parental tumor and PDT-CUP#1 tumoroids.

To validate representative sequence variants identified using FoundationOne® CDx, Sanger sequencing was performed. *STK11* (p.D194N), *TP53* (p.R175H and p.R213Q), and *MAP3K1* (p.S941*) mutations were detected in both PDT-CUP#1 tumoroids and the parental tumor. In contrast, the *TERT* promoter mutation (-124C>T) detected by FoundationOne® CDx was not identified in genomic DNA extracted from PDT-CUP#1 tumoroids (Fig. 3d–g). These findings indicate that PDT-CUP#1 retains the major genomic features of the parental tumor while preserving its principal driver sequence variants. We also performed STR profiling of PDT-CUP#1 to facilitate authentication and assessment of genomic stability during long-term passaging (Fig. S5).

### Comparison of pathological features in parental tumor and the xenograft derived from PDT-CUP#1 tumoroids

**Fig. 4:**
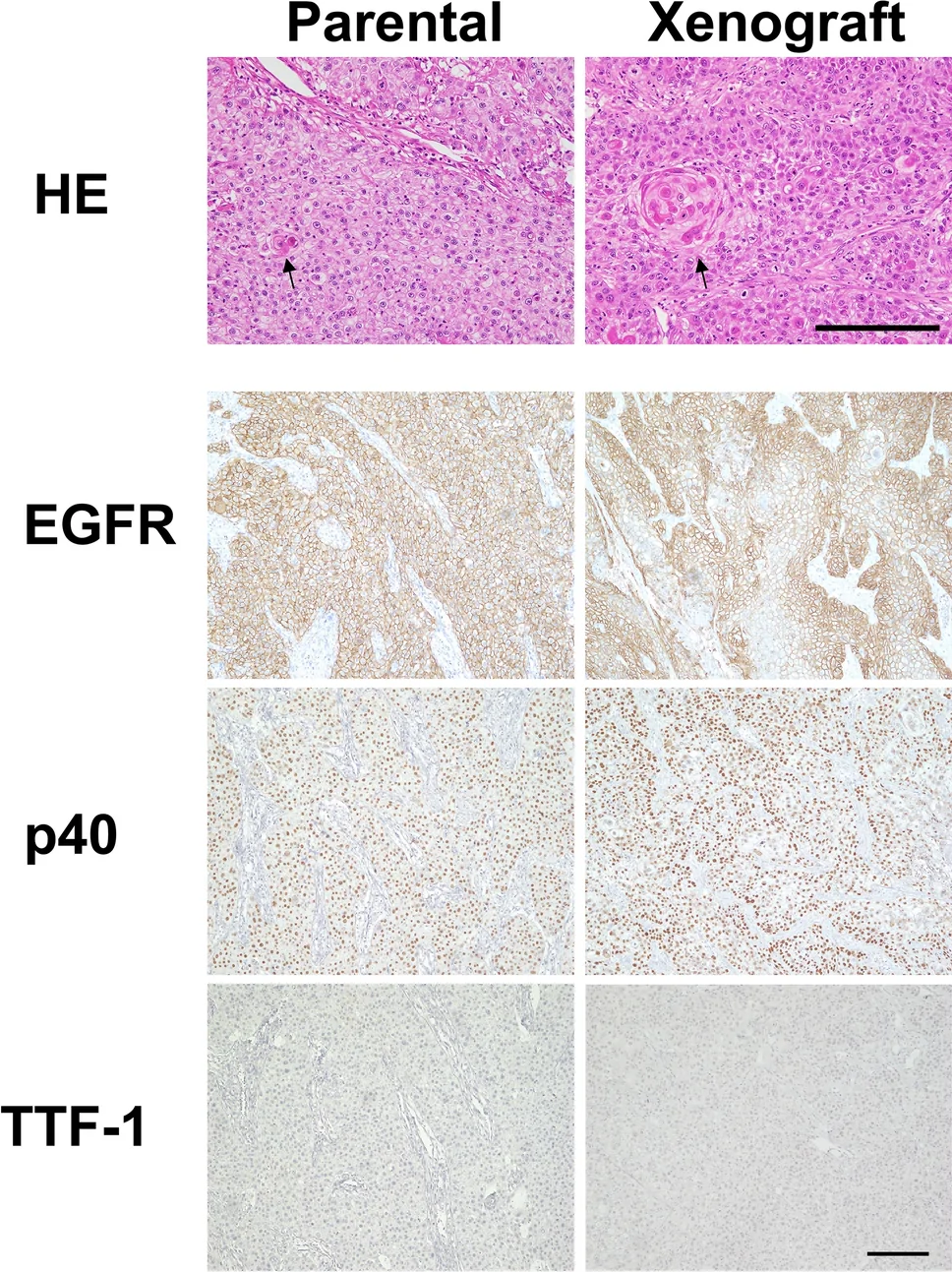
Pathological findings of the primary tumor and xenograft derived from PDT-CUP#1 tumoroids. H and E staining revealed the presence of both undifferentiated and differentiated squamous cell carcinoma components. Similar histological features were observed in the xenograft tumors. The arrows indicate the keratin pearls. Robust cytoplasmic EGFR expression was detected in both primary tumors and xenografts. Nuclear p40 expression was confined to the differentiated squamous cell carcinoma component in both the primary tumor and xenograft, whereas nuclear TTF-1 expression was minimal in both specimens. Scale bars: 200 μm

To analyze the pathological features of the PDT-CUP#1 tumoroids, xenografts were established in NOD.Cg*-Prkdc*^*scid*^* Il2rg*^*tm1Wjl*^*/SzJ* (NSG) mice and their histopathology was directly compared with that of the parental tumor. As shown in Fig. 4, hematoxylin and eosin (H and E) staining of the parental tumor revealed differentiated squamous cell carcinoma, characterized by abundant keratinizing cells, and undifferentiated squamous cell carcinoma. Similar histological features were observed in the xenograft tumor. IHC revealed strong EGFR expression along the cytoplasmic membrane in both parental tissue and xenograft. In addition, nuclear p40 expression was detected in both tissues, whereas TTF-1 expression was absent in either.

### PDT-CUP#1 tumoroids demonstrated sensitivity against erlotinib, cetuximab, necitumumab, and depatuxizumab mafodotin

**Fig. 5:**
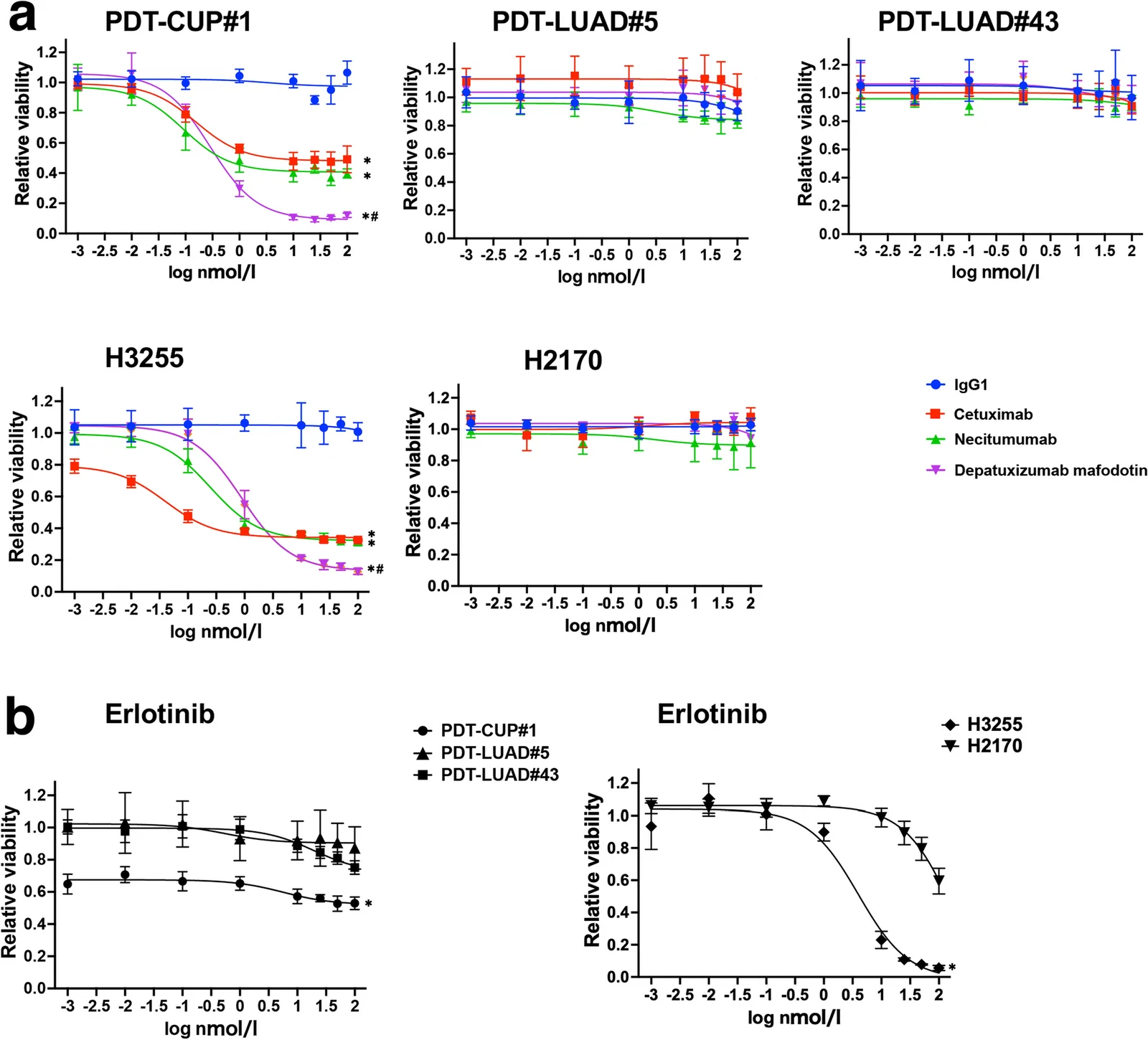
Sensitivity of PDT-CUP#1 tumoroids to erlotinib, cetuximab, necitumumab, and depatuxizumab mafodotin. **a** Cetuximab and necitumumab moderately suppressed the viability of PDT-CUP#1 tumoroids, whereas depatuxizumab mafodotin induced a marked reduction in viability after 96 h. In contrast, PDT-LUAD#5 and PDT-LUAD#43 tumoroids and H2170 lung squamous cell carcinoma cells exhibited minimal responses to these therapeutic agents. Human IgG1 was used as the non-targeting isotype control. H3255 pulmonary adenocarcinoma cells harboring an activating *EGFR* exon 21 L858R mutation with concomitant *EGFR* amplification were sensitive to these drugs. **b** Erlotinib weakly suppressed the viability of PDT-CUP#1 tumoroids compared with that of PDT-LUAD#5 and PDT-LUAD#43 tumoroids. In contrast, H3255 pulmonary adenocarcinoma cells were highly sensitive to erlotinib, consistent with the presence of *EGFR* amplification and an *EGFR* exon 21 L858R mutation. *p < 0.01 versus the corresponding human IgG1 isotype control or H3255 cells. ^#^*p* < 0.01 compared with necitumumab

To identify potential therapeutic agents for patients with CUP, we performed a drug sensitivity assay to evaluate the responsiveness of patient-derived tumoroids to four therapies targeting the EGFR: erlotinib, a tyrosine kinase inhibitor (TKI); the monoclonal antibodies cetuximab and necitumumab; and depatuxizumab mafodotin, an antibody–drug conjugate (ADC) carrying monomethyl auristatin F. Cetuximab is clinically approved for EGFR-expressing colorectal and head and neck cancers [27, 28], whereas necitumumab is approved for squamous non-small cell lung cancer [29]. At 96 h after treatment, cetuximab and necitumumab significantly suppressed the growth of PDT-CUP#1 tumoroids compared with their effects on PDT-LUAD#5 and PDT-LUAD#43 tumoroids (Fig. 5a, Table S4). qPCR analysis demonstrated that PDT-CUP#1 tumoroids showed the second-highest transforming growth factor alpha (TGFA) mRNA expression, following PDT-LUAD#43. PDT-CUP#1 also exhibited higher amphiregulin (AREG) mRNA expression than PDT-LUAD#5 and PDT-LUAD#43 (Fig. S6). These expression levels were comparable to those observed in A549 pulmonary adenocarcinoma cells, in which detectable expression of TGFA and AREG, both encoding ligands for EGFR, has been reported in the Human Protein Atlas [30]. In addition, depatuxizumab mafodotin, originally developed for *EGFR-*amplified glioblastoma [31], most clearly inhibited the growth of PDT-CUP#1 tumoroids at 96 h after treatment. However, it did not demonstrate significant antitumor effects against other tumoroid types. H3255 pulmonary adenocarcinoma cells, which harbor *EGFR* amplification and exhibit the second-highest level of EGFR expression after PDT-CUP#1, were also sensitive to cetuximab, necitumumab, and depatuxizumab mafodotin. In contrast, H2170 lung squamous cell carcinoma cells, which lack *EGFR* amplification, showed minimal responses to these EGFR-targeted agents. This cell line harbored an *EGFR* exon 21 L858R mutation besides *EGFR* amplification and showed greater sensitivity to the EGFR-TKI erlotinib than PDT-CUP#1 tumoroids, which harbored *EGFR* amplification without an activating mutation (Fig. 5b). Erlotinib, which is used not only for non-small cell lung cancers harboring *EGFR* mutations but also for those with EGFR overexpression [32, 33], more effectively suppressed the viability of PDT-CUP#1 tumoroids than that of PDT-LUAD#5 and PDT-LUAD#43 pulmonary adenocarcinoma tumoroids, which exhibited lower EGFR expression, at 96 h after treatment. H3255 pulmonary adenocarcinoma cells were highly sensitive to erlotinib, consistent with the presence of *EGFR* amplification and an *EGFR* exon 21 L858R mutation. In contrast, H2170 showed minimal responses to these EGFR-targeted agents.

### Clinical course and response to platinum-based chemotherapy combined with necitumumab

**Fig. 6:**
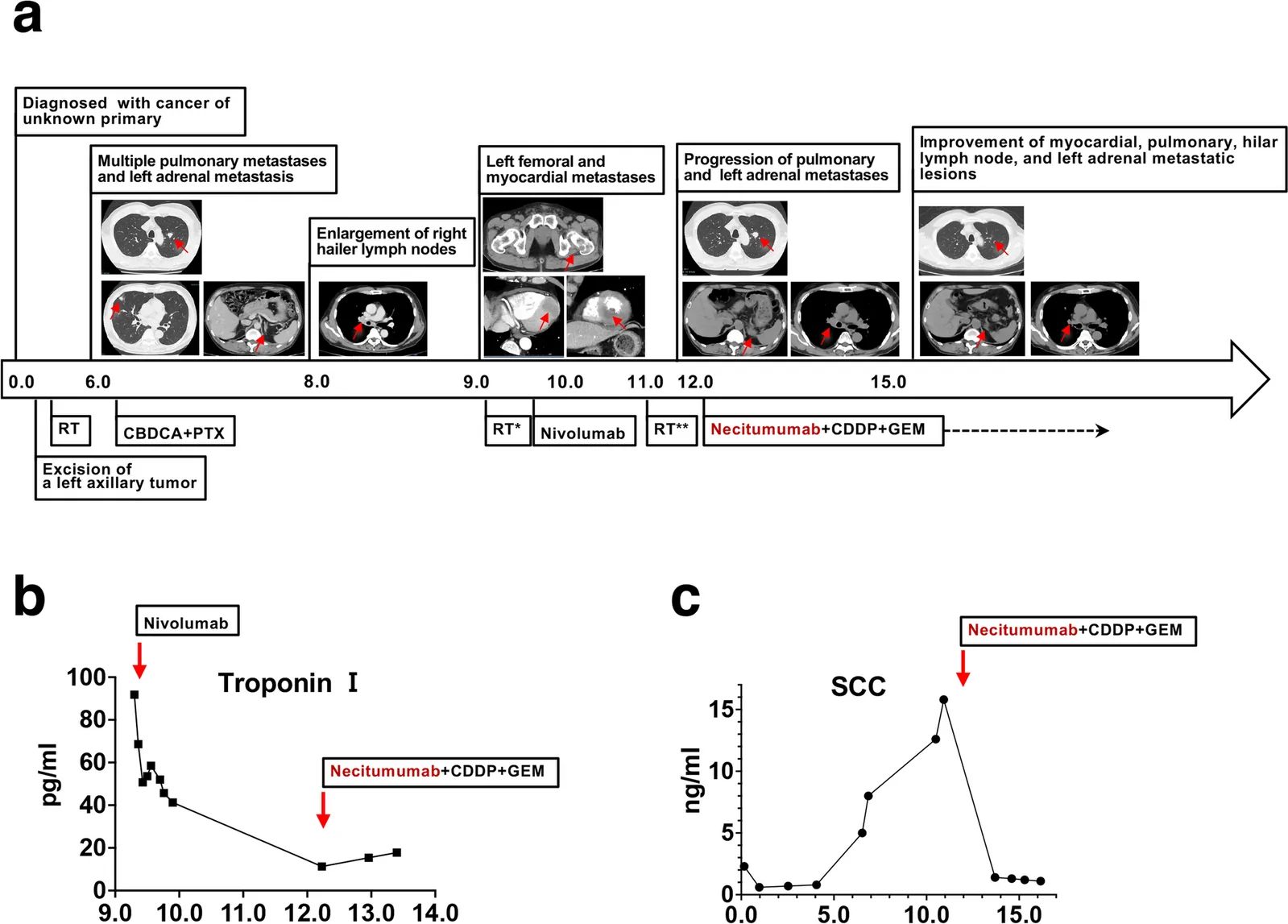
Clinical validation of necitumumab efficacy predicted via tumoroid testing. **a** Timeline of treatment for a patient with CUP harboring *EGFR* amplification. Numbers indicate time (mo) since the initial diagnosis. CBDCA; carboplatin, PTX; paclitaxel, CDDP; cisplatin, GEM; gemcitabine, RT* palliative radiation therapy for femoral metastases, RT** radiation therapy for bronchial invasion caused by right hilar lymph node metastases and for control of endobronchial bleeding. **b** Serum troponin I levels decreased after nivolumab treatment. **c** Squamous cell carcinoma antigen (SCC) levels decreased after treatment with necitumumab in combination with platinum-based chemotherapy

In a patient with CUP, the EGFR-targeting monoclonal antibody necitumumab was administered in combination with platinum-based chemotherapy following disease progression after multiple prior treatments. The clinical course is depicted in Fig. 6a**.** At diagnosis, the patient was diagnosed with CUP. One mo after diagnosis, surgical excision of the left axillary tumor was performed, followed by radiotherapy. At six mo after diagnosis, multiple pulmonary metastases and a left adrenal metastasis were detected, and first-line chemotherapy with carboplatin plus paclitaxel was initiated. Following disease progression after carboplatin plus paclitaxel chemotherapy, nivolumab was administered to treat CUP. Although the myocardial metastasis regressed, nivolumab treatment was discontinued because of hepatotoxicity. Thereafter, the pulmonary and left adrenal metastases progressed. Immunohistochemical analysis of the parental tumor performed in the present study demonstrated high PD-L1 expression (tumor proportion score [TPS] >50%, Fig. S7), despite the limited overall clinical benefit from nivolumab. Consequently, platinum-based chemotherapy combined with necitumumab was initiated 12 mo after diagnosis. By 16 mo after diagnosis, improvements were observed in the myocardial, pulmonary, hilar lymph node, and left adrenal metastases. The myocardial enzyme troponin I, which had increased nine mo after diagnosis, decreased following nivolumab treatment (Fig. 6b). In contrast, the tumor marker squamous cell carcinoma antigen (SCC) continued to increase during nivolumab therapy. SCC levels markedly decreased after the initiation of platinum-based chemotherapy combined with necitumumab, whereas troponin I levels remained low (Fig. 6c), suggesting that platinum-based chemotherapy combined with necitumumab was clinically effective in this patient.

## Discussion

CUP is associated with a poor prognosis, and treatment strategies based on identification of the primary site or molecular alterations remain incompletely established [34]. Although recent preclinical studies using CUP-derived cancer models have begun to demonstrate the feasibility of functional precision oncology, clinically applicable frameworks remain limited [35–37].

Although an occult pulmonary primary cannot be completely excluded, no pulmonary lesion was identified using contrast-enhanced CT or PET-CT at diagnosis, and therefore this case fulfilled the current clinicopathological diagnostic criteria for CUP. Furthermore, no histological evidence supporting a primary cutaneous squamous cell carcinoma, including epidermal dysplasia, carcinoma in situ, or continuity between the epidermis and the tumor, was identified. These clinicopathological findings support the diagnosis of CUP.

Recent advances in molecular diagnostic technologies have also expanded the diagnostic evaluation of CUP. Gene expression signature assays and tissue-of-origin analyses have demonstrated utility in predicting tumor origin and distinguishing carcinomas arising from different anatomical sites [38, 39]. In particular, 90 gene expression profiling has been reported to improve tissue-of-origin prediction in CUP cases. Although these approaches were not performed in this study, they may provide complementary information to conventional clinicopathological evaluation and genomic profiling and could further support diagnostic classification in challenging cases.

While case reports have described favorable responses to EGFR-TKIs in CUP harboring activating *EGFR* mutations [40, 41], therapeutic outcomes in *EGFR-*amplified CUP have not been well characterized. Here, we established a CUP-derived tumoroid model and performed functional drug testing to evaluate differential sensitivity across EGFR-targeted modalities.

Although *EGFR* amplification was identified as a candidate driver alteration via comprehensive genomic profiling, this information alone did not indicate the most effective candidate class of EGFR-targeted therapy. Functional testing using patient-derived tumoroids demonstrated modest sensitivity to EGFR-TKIs, moderate growth inhibition by anti-EGFR monoclonal antibodies, and pronounced antitumor activity of an EGFR-targeted ADC. These findings demonstrate that therapeutic sensitivity among EGFR-targeted agents varies depending on drug modality, even when targeting the same genomic alteration.

Although erlotinib demonstrated greater growth suppression in PDT-CUP#1 compared with low-EGFR–expressing tumoroids, its activity remained modest relative to that of antibody- and ADC-based strategies. Importantly, *EGFR* amplification and EGFR-activating mutations represent biologically distinct genomic events. Activating mutations often confer kinase dependency and robust sensitivity to TKIs, whereas gene amplification represents a quantitative increase in receptor copy number and may not necessarily establish dominant kinase-driven signaling. This distinction could probably be one of the reasons why erlotinib exhibited only modest growth suppression in PDT-CUP#1, despite higher EGFR expression compared with that in control tumoroids and relatively higher sensitivity compared with low-EGFR–expressing models.

The differential efficacy observed among EGFR-targeted therapies may reflect mechanistic differences between drug classes. EGFR-TKIs primarily inhibit kinase activity and are generally more effective in tumors harboring activating *EGFR* mutations [42]. In contrast, anti-EGFR monoclonal antibodies inhibit ligand binding and receptor activation [43, 44]. In H3255 cells harboring an *EGFR* exon 21 L858R mutation, anti-EGFR antibodies interfere with mutation-dependent receptor activation [45]. In contrast, PDT-CUP#1 did not harbor activating *EGFR* mutations. Given the detectable expression of EGFR ligands including TGFA and AREG in PDT-CUP#1 (Fig. S6), the observed antitumor effects of cetuximab and necitumumab may be attributable to ligand-dependent EGFR signaling. Thus, although cetuximab and necitumumab demonstrated antitumor activity in both H3255 cells and PDT-CUP#1 tumoroids, the underlying molecular mechanisms are likely distinct in these models.

Depatuxizumab mafodotin demonstrated the most pronounced growth-inhibitory effect in vitro. As an EGFR-targeted antibody–drug conjugate, its antitumor activity is mediated by delivery of a cytotoxic payload rather than by inhibition of EGFR signaling alone [46]. Although clinical development of this agent was limited by toxicity [47], advances in ADC technology may offer improved therapeutic options for *EGFR*-amplified tumors in future.

The sensitivity to necitumumab was notably concordant with radiological tumor regression and a reduction in SCC tumor marker levels following platinum-based chemotherapy combined with necitumumab in the corresponding patient. Although a definitive causal relationship cannot be established from a single case, the observed concordance supports the potential translational relevance of tumoroid-based drug testing. As this study was based on a single case, the findings should be interpreted cautiously. Another limitation is that we did not compare the genomic profiles of early and late-passage tumoroids. Therefore, although WES demonstrated substantial genomic concordance between the parental tumor and PDT-CUP#1, WES alone cannot exclude the possibility that additional sequence alterations accumulated during serial in vitro propagation. Previous studies have suggested that organoid cultures better preserve tissue characteristics than conventional two-dimensional cultures [48]. In addition, in our previous study of patient-derived esophageal tumoroids, the major pathogenic mutations detected at early passages were retained after long-term culture [10]. Nevertheless, longitudinal genomic analyses across serial passages will be required to directly evaluate culture-associated genomic evolution in future studies.

Beyond this individual case, our results illustrate how patient-derived tumoroids can generate functional information complementary to genomic profiling. Importantly, this study provides proof-of-concept evidence that functional drug testing may aid therapeutic prioritization in CUP. Copy number alterations, including gene amplifications, are frequently detected across cancer types; however, their therapeutic implications are often context-dependent. These findings suggest that copy number–driven oncogenic alterations may benefit from functional validation when selecting optimal drug modalities, even within a single target pathway. Tumoroid-based functional assessment may therefore assist in therapeutic prioritization in selected patients with CUP and other clinically complex malignancies. Further validation in larger cohorts will be necessary to define the broader clinical applicability of this approach.

## Supplementary Information

Below is the link to the electronic supplementary material.

Supplementary file1 (PDF 4577 KB)

Supplementary file2 (XLSX 21 KB)

## Data Availability

PDT-CUP#1 tumoroids will be distributed by the RIKEN Cell Bank after publication of this study (https://cell.brc.riken.jp/en/). The whole-exome sequencing dataset has been submitted to the Japanese Genotype-phenotype Archive (JGA) for controlled access. Updated information on the dataset and its accession number will be available through the NBDC Human Database under the research ID hum0419. All remaining data are available from the authors upon reasonable request, except for patient-identifying information.
