# Supplementary material for: Establishment and functional characterization of an EGFR-amplified tumoroid from squamous cell carcinoma of unknown primary
Source: Hum Cell. 2026 Aug 19;39(9):127. doi: 10.1007/s13577-026-01440-x (PMC13490018; doi:10.1007/s13577-026-01440-x)
Supplement: Supplementary file 1 [file 13577_2026_1440_MOESM1_ESM.pdf]

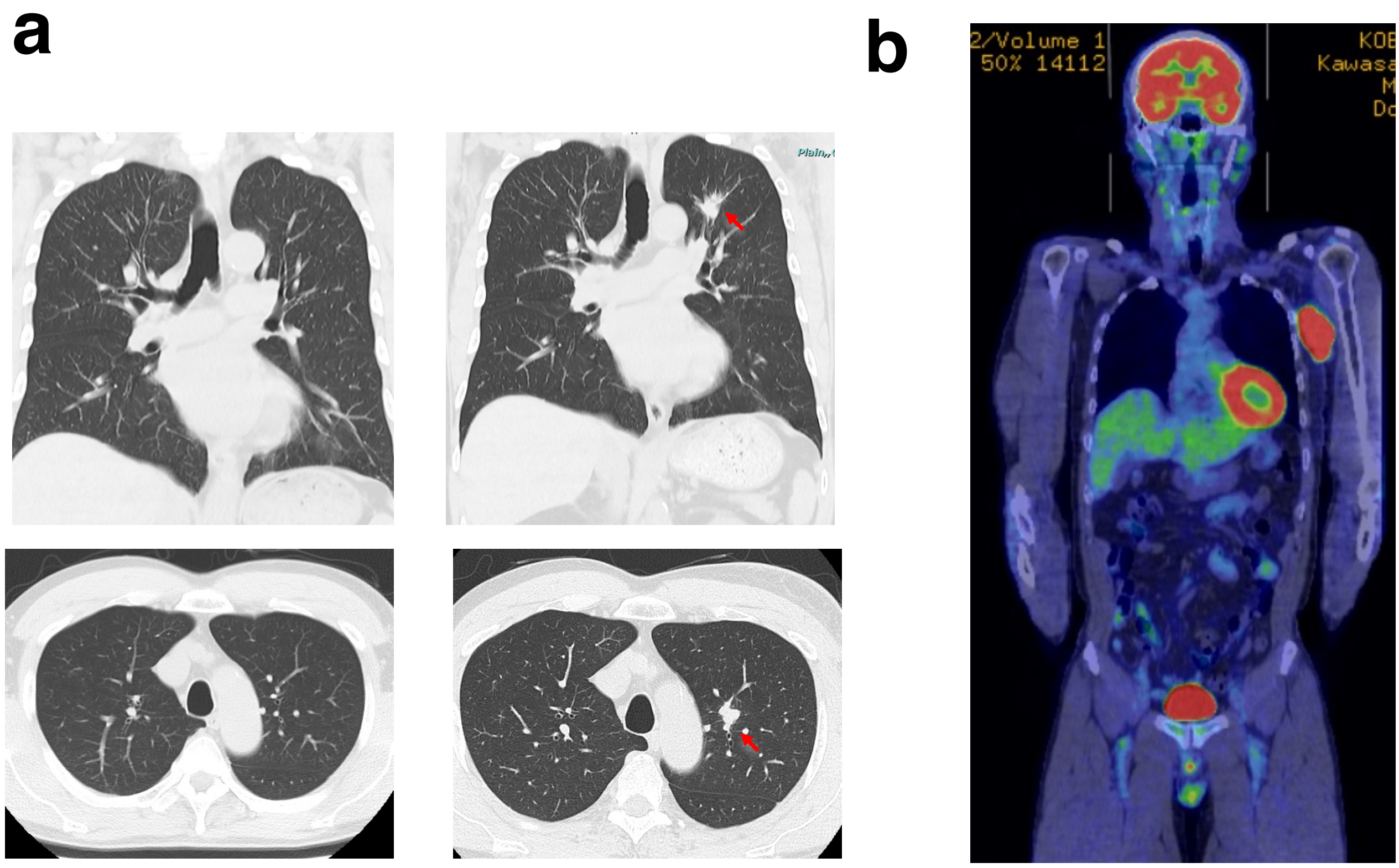

Fig. S1 Pulmonary findings on preoperative examination.

(a) No pulmonary nodules were detected on preoperative chest computed tomography (CT) scan; however, a pulmonary nodule was identified six mo after the initial visit (red arrows, right columns). (b) Positron emission tomography–computed tomography (PET-CT) showed no abnormal FDG uptake other than in the axillary lesion.

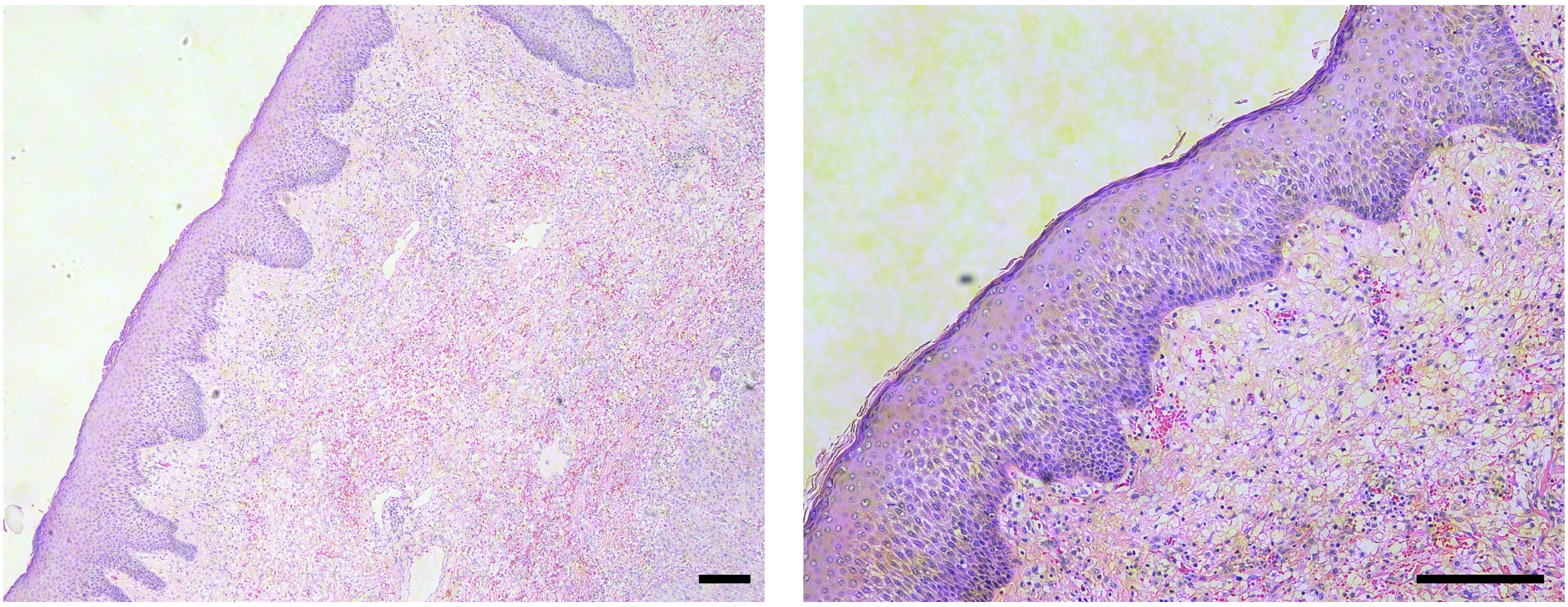

Fig. S2 Evaluation of skin involvement in the left axillary mass.

Histopathological examination of the resected left axillary mass did not provide any evidence of epidermal dysplasia or carcinoma *in situ*. In addition, no histological continuity between the tumor and epidermis was observed, ruling out a cutaneous origin. Scale bars: 200 μm.

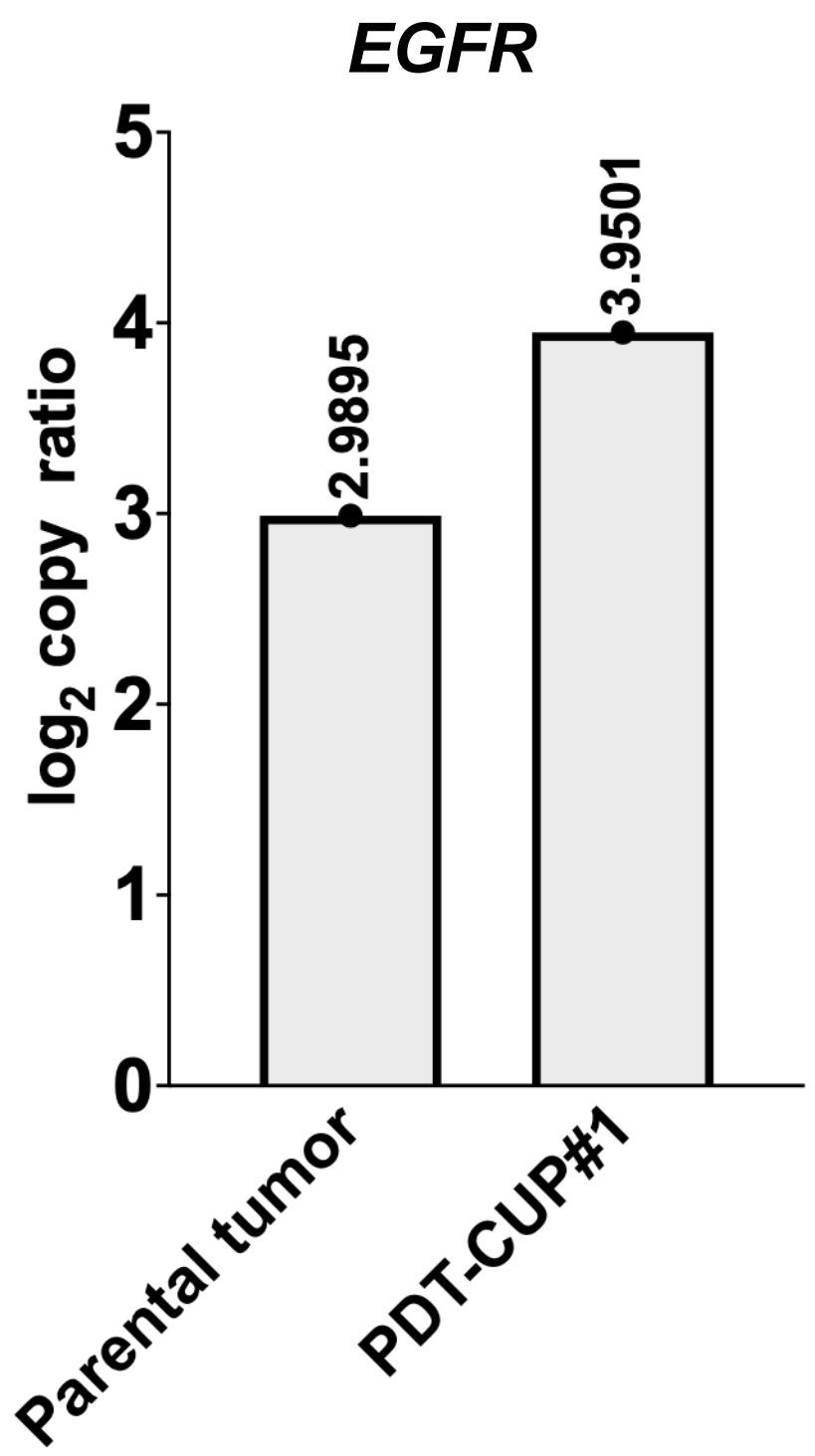

Fig. S3 Whole-exome sequencing-based copy number analysis of *EGFR* in the parental tumor and PDT-CUP#1 tumoroids.

Whole-exome sequencing (WES)-based copy number analysis demonstrated high-level *EGFR* amplification in both the parental tumor and the corresponding PDT-CUP#1 tumoroids. The estimated $\log_2$ copy ratios were 2.99 and 3.95, respectively, indicating that *EGFR* amplification was retained during tumoroid establishment.

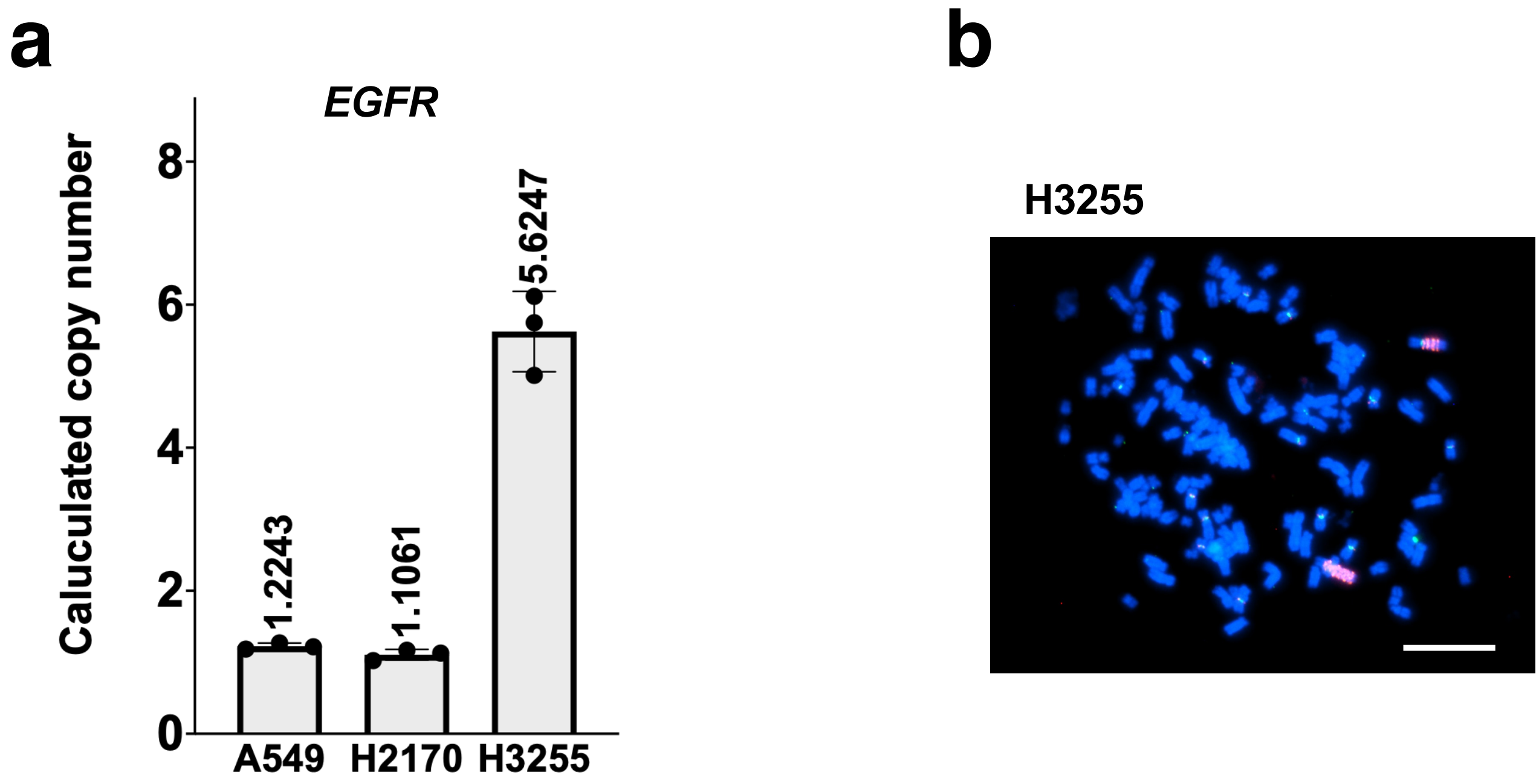

Fig. S4 *EGFR* amplification in H3255 pulmonary adenocarcinoma cells.

a) qPCR analysis revealed *EGFR* amplification in H3255 pulmonary adenocarcinoma cells. b) Metaphase FISH demonstrated amplification of *EGFR,* located on chromosome 7, in H3255 cells. Scale bar: 20 μm.

a

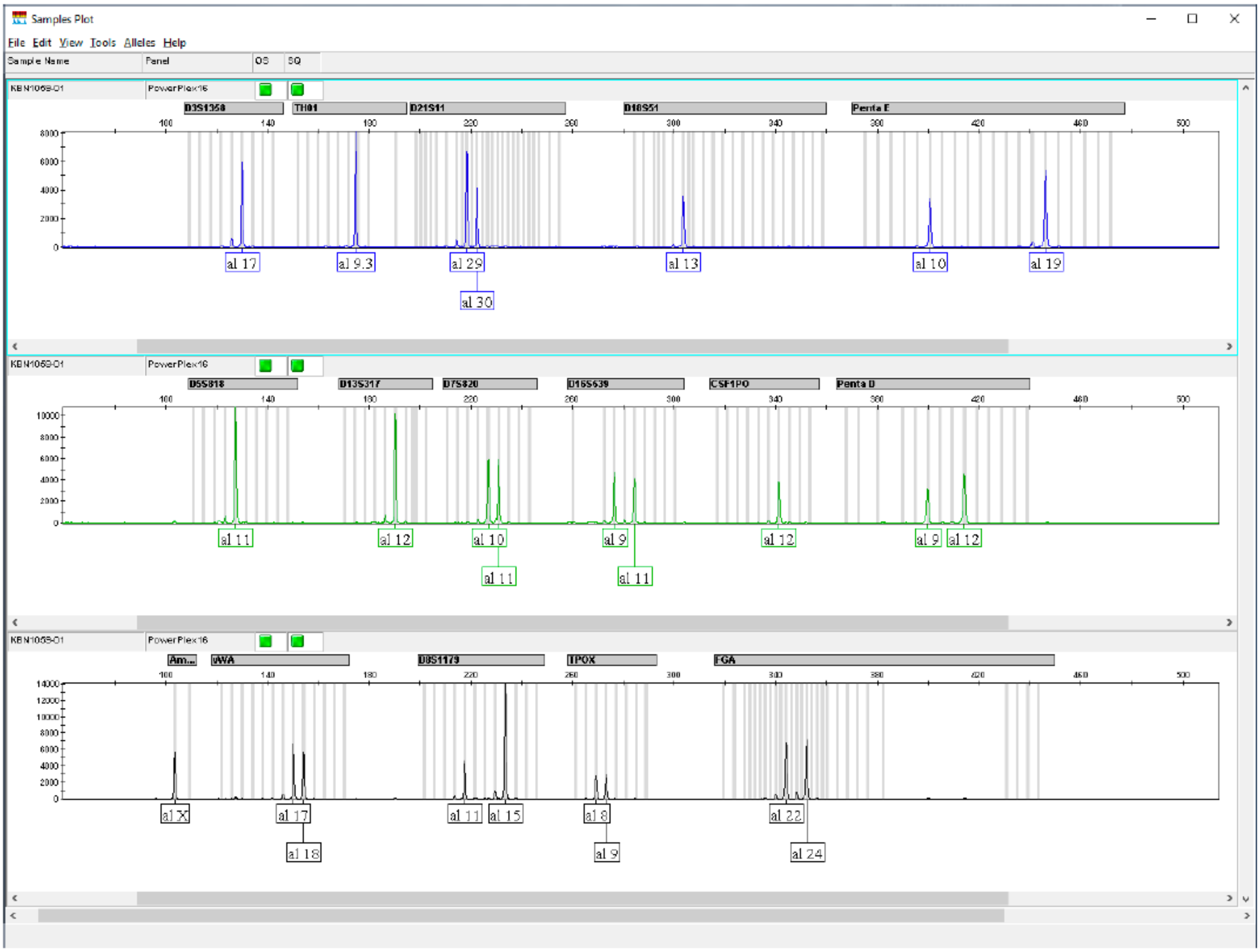

b

D3S1358	TH01	D21S11	D18S51	Penta_E
17	9.3	29,30	13	10,19

D5S818	D13S317	D7S820	D16S539	CSF1PO	Penta_D
11	12	10,11	9,11	12	9,12

AM	vWA	D8S1179	TPOX	FGA
X	17,18	11,15	8,9	22,24

Fig. S5 STR profile analysis of PDT-CUP#1 tumoroids.

Representative peak report (a) and summarized STR profile (b) of PDT-CUP#1.

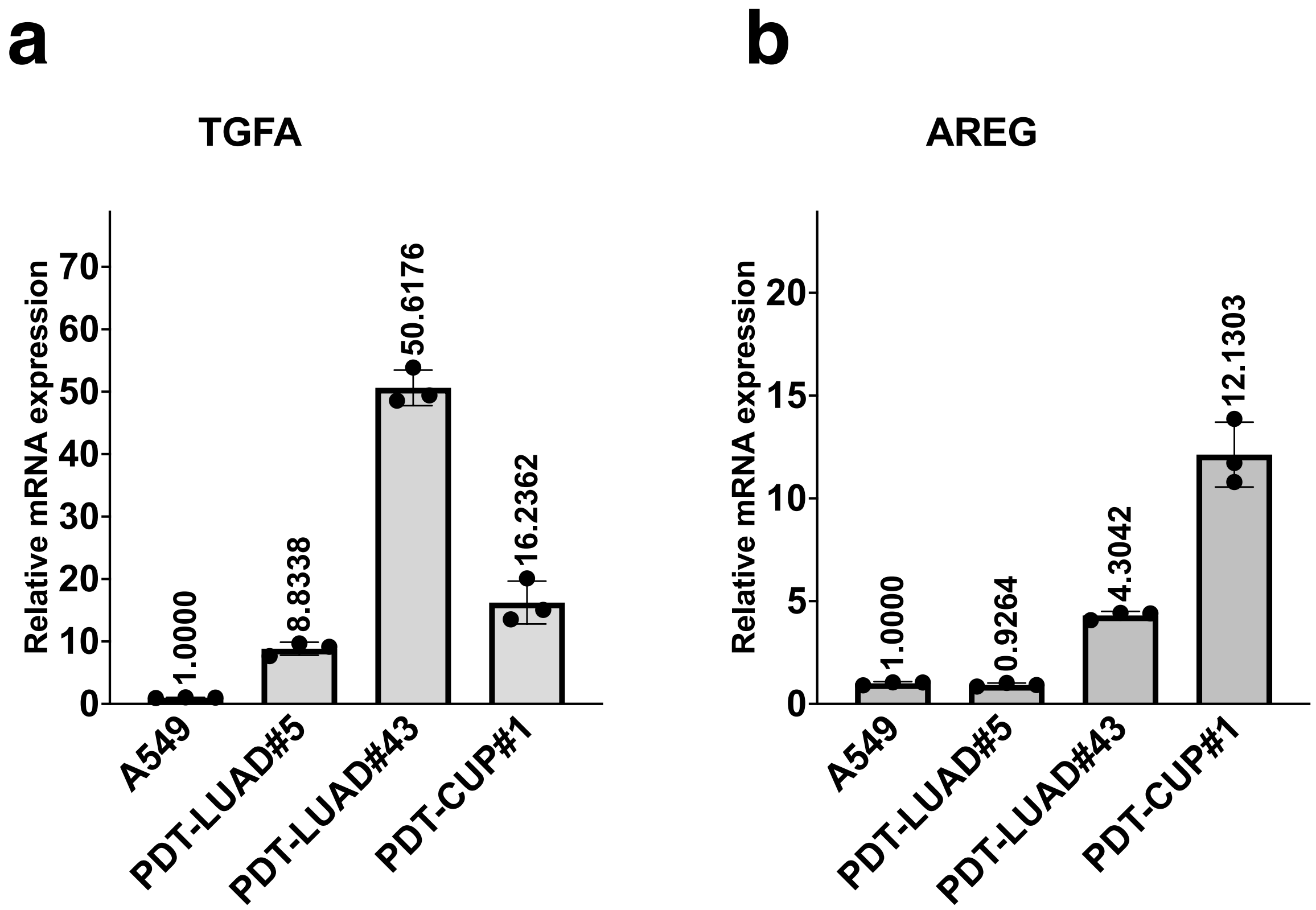

Fig. S6 Detection of TGFA and AREG mRNA expression in PDT-CUP#1 tumoroids.

a) TGFA and b) AREG mRNA expression levels were quantified via qPCR using specific TaqMan probes (TGFA, Hs00608187_m1; AREG, Hs00950669_m1). Expression levels were normalized to those in A549 pulmonary adenocarcinoma cells, which were set to 1.0.

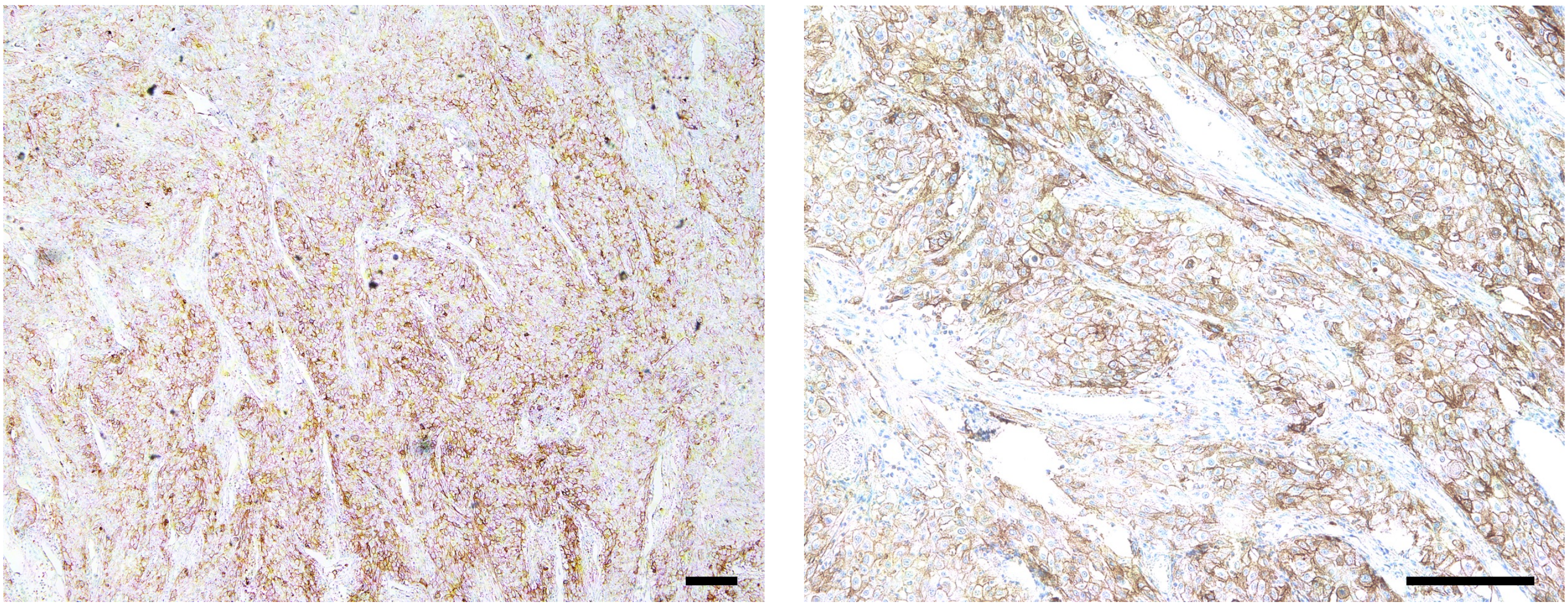

Fig. S7 Immunohistochemical evaluation of PD-L1 expression in the metastatic lymph node lesion.

Immunohistochemical staining using the 22C3 pharmDx assay revealed membranous PD-L1 expression in tumor cells within the metastatic lymph node lesion. The tumor proportion score (TPS) was greater than 50%. Scale bars: 200 μm.
